# To Be Is To Become. Fractal Neurodynamics of the Body-Brain Control System

**DOI:** 10.3389/fphys.2020.609768

**Published:** 2020-12-15

**Authors:** Franca Tecchio, Massimo Bertoli, Eugenia Gianni, Teresa L'Abbate, Luca Paulon, Filippo Zappasodi

**Affiliations:** ^1^Laboratory of Electrophysiology for Translational NeuroScience, Institute of Cognitive Sciences and Technologies-Consiglio Nazionale delle Ricerche, Rome, Italy; ^2^Department of Neuroscience, Imaging and Clinical Sciences, University ‘Gabriele d'Annunzio’ of Chieti-Pescara, Chieti, Italy; ^3^Unit of Neurology, Neurophysiology, Neurobiology, Department of Medicine, Università Campus Bio-Medico di Roma, Rome, Italy

**Keywords:** plasticity, synchrony, feedback, neurodynamics, recursive multiscale triadic principle

The Network Physiology field frames the multi-scale multi-dimensional nature of the body system emerging in the interaction among organs, which interplay via hemodynamic and metabolic functions under hormonal and neuronal controlling communication (Bashan et al., 2012; Ivanov and Bartsch, 2014; Bartsch et al., 2015; Ivanov et al., 2016; Lin et al., 2016). Thus, while the Network Physiology models networks consisting of organs (nodes) that are heterogeneous and connected by systems (connectors) of a still different nature, the brain is made up of elements that are at the same time nodes (soma of the neuron) and connectors (axons), so that the communicative—necessary and sufficient—nature confers to the sets of neurons the status of Network. Here we refer to Neuronal Networks [NN], which structurally include at least one node receiving inputs from the environment, and one node producing outputs to the environment; the NN connections are necessarily both negative and positive; every NN's node “necessarily” produces a pattern-OUT when the pattern-IN arrives, overall resulting in a specific local time course of the electrical neuronal activity, the local neurodynamics.

Here, grounding on existing knowledge, we propose a unique functional organizing principle—the feedback-synchrony-plasticity triad—which, governing the neuronal networks at multiple scales, emerges as a potential explanatory framework for the fractal properties exhibited by neurodynamics. In a translational perspective, via the strategy of “listening” to the body-brain organization by non-invasive electrophysiological techniques (electro- and magneto-encephalography and electromyography) integrated with “intervening” by non-invasive brain stimulation techniques, we exploited the communication means used by neuronal networks to enhance the capability of fighting symptoms secondary to neurodynamics dysfunctions. In other words, we introduce precision approaches to electroceuticals, i.e., the cure of ailments by means of electrical signals (Reardon, 2014).

## The Feedback-Synchrony-Plasticity Triadic Principle (FeeSyCy) Governs the Body-Brain System

We consider the whole brain as a neurons' ensemble which coordinates the interaction of the body brain network with the environment, where input depends on the output and the other way round, the output depends on the input, working in a feedback loop. Via somatic, proprioceptive (Rossi et al., 1998; Fink et al., 2014), visual and auditory sensory receptors, our motor actions produce from the environment feedback, that our brain shapes dependently on the desired goal (Friston, 2018). This feedback loop stimulates our brain neurons inducing locally specific dynamic synchronizations among the nodes of dedicated functional networks (Tecchio et al., 2008; Gandolla et al., 2014). Such synchronizations within the network's subsystems imply a desynchronization of those very subsystems with the wider regions they are part of, resulting in a reduction of the resting-state high power of the cortical activity paced within the thalamocortical loops (Gent et al., 2018), e.g., alpha reactivity (Klimesch, 1999). In turn, these modulations of synchrony engage the system in adaptations either sustaining the execution as planned or enabling proper corrections (Fink et al., 2014). In this process, our neurons implement output changes following a key rule (Kandel and Schwartz, 1985): if two input signals reach the neuron together, the neuron increases its probability to fire (Hebb, 1949), that is to produce an action potential transmitting a message. Some authors indicate that the Hebbian rule subtends main trial-and-error (Hoerzer et al., 2014) and imitation (Keysers and Gazzola, 2014) learning mechanisms. This continuous adaptation capability shapes the ability of our neurons to change their output according to what is required, quantified depending on the distance between the expected outcome and the current one. When the distance is small, behavioral adaptations emerge through the current network setup [working adaptation (Wolpert et al., 2011)]. When the distance is big, new skill acquisitions emerge through even huge structural changes (plastic adaptation, i.e., learning). A richness and complexity of molecular and cellular phenomena and of signaling, in continuous discovery, underlie the cellular and network modifications that implement the plastic adaptations. Plasticity mechanisms occurring at the synapses' level with non-unitary interplaying potentiation and depression phenomena (Malenka and Bear, 2004) are integrated by intrinsic plasticity mechanisms (Zhang and Linden, 2003) and changes in myelin multi-laminar sheaths that modulate the timing of information transmission between relay points through neural circuits, inducing changes in spike arrival-time, with which a high degree of precision controls the probability of activation (Gibson et al., 2014; Fields, 2015). It is supposed that Hebbain rules acting in day time, are supported during sleep spontaneous activity, by renormalizations of net synaptic strengths (Tononi and Cirelli, 2014) implementing homeostatic plasticity (Turrigiano and Nelson, 2004).

Notably, the feedback-synchrony-plasticity (*FeeSyCy*) triadic principle that governs motor control, controls the whole body-brain system. We can recognize some paradigmatic examples of the breakup of one of the three links in the FeeSyCy chain, which generates the breakup of the whole process.

### Feedback Link Breakup

The lack of auditory training and feedback condemned for centuries deaf individuals, despite owning intact motor executive functionality, to the inability to develop linguistic production, that is it condemned them to live as a deaf-mute (Sacks, 1989). The role of feedback is strongly proven by deaf people who grow nowadays. Starting from the last century, the teaching models and techniques -guided by neuroscientific comprehension–have definitely revolutionized the condition of deaf people, who now can, in parallel to the sign language, achieve an excellent production of language vocal expression by exploiting during their development the feedback about their produced words properly translated in signals from the spared sensory channels, mainly the visual one.

### Synchrony Link Breakup

In dystonic individuals, despite proper sensory stimuli being transmitted *via* intact sensory systems, the impaired intracerebral synchronizations subtending the sensorimotor integration (Melgari et al., 2013), impairs the motor control (Abbruzzese and Berardelli, 2003).

### Plasticity Link Breakup

Schizophrenic individuals are able to move and receive proper sensory feedback from the environment but cannot engage in proper adaptation due to neuronal inability to involve the metabolic chains and adapt the cells *via* plasticity (Ramocki and Zoghbi, 2008).

## The FeeSyCy Triadic Principle Manifests Itself Recursively at Multiple Scales

### Single Neurons' Network

In *in-vitro* primary cell culture of single cortical pyramidal neurons of postnatal rats, the synaptic changes implementing long-term potentiation and depression emerged as a function of incoming activity (Turrigiano et al., 1998; Sjöström and Nelson, 2002). Synaptic potentiation increases the postsynaptic firing rates in correlation with presynaptic activity, producing a positive feedback loop. Multiplicative scaling of synaptic strengths preserves relative differences between inputs, allowing a non-saturated implementation of Hebbian modifications (Hebb, 1949).

### Neuronal Pools' Network

In functioning of multiple brain areas networks, a parallel capturing of bottom-up patterns of activation in sensory-motor areas occurs together with a top-down processing that selects sensory-motor activations to implement long-lasting storage. As memories organize themselves in central structures, they implement an active selection of sensory experience, proprioception and emotional knowledge for further learning (Barsalou, 1999).

### Body-Brain Network

Deepening the paradigmatic example of motor execution, skilled actions require the actual gathering of sensory information, which is processed extracting what is relevant to the planned action. Such feedback comes from different types of information that the motor system uses as a learning signal, including error-based, reinforcement, observational and use-dependent information. In all cases, motor learning occurs implementing adaptations dependent on the distance between the expected and occurring inputs (Wolpert et al., 2011).

We can recognize an expression at the whole system level of the multi-scale recursive FeeSyCy principle in the human gait showing fractal dynamics (Hausdorff et al., 1996; Phinyomark et al., 2020) and also across species, in experimental data about food-searching strategies in insect, mammal and bird species (Edwards et al., 2007).

## Working at Multiple Scales, the FeeSyCy Principle Subtends a Fractal Neurodynamics

When a system presents the whole structure that is made up of single blocks, which are similar to the whole, and are in turn made of smaller blocks, similar to it and to the whole structure, it is a fractal. Its name comes from a non-integer number that quantifies its dimension. In our case, FD estimates on a time window the distance between the amplitudes of successive neuronal electrical activity points, in relationship with the time sampling.

Brain neurodynamics displays the so-called “power law” (He, 2011), i.e., the power of the signal generated by a neuronal population follows an exponential behavior. Among the multiple signals with a spectrum that distributes as power law, we propose the hypothesis that brain signals are fractal (Buzsaki and Mizuseki, 2014).

**Figure 1:**
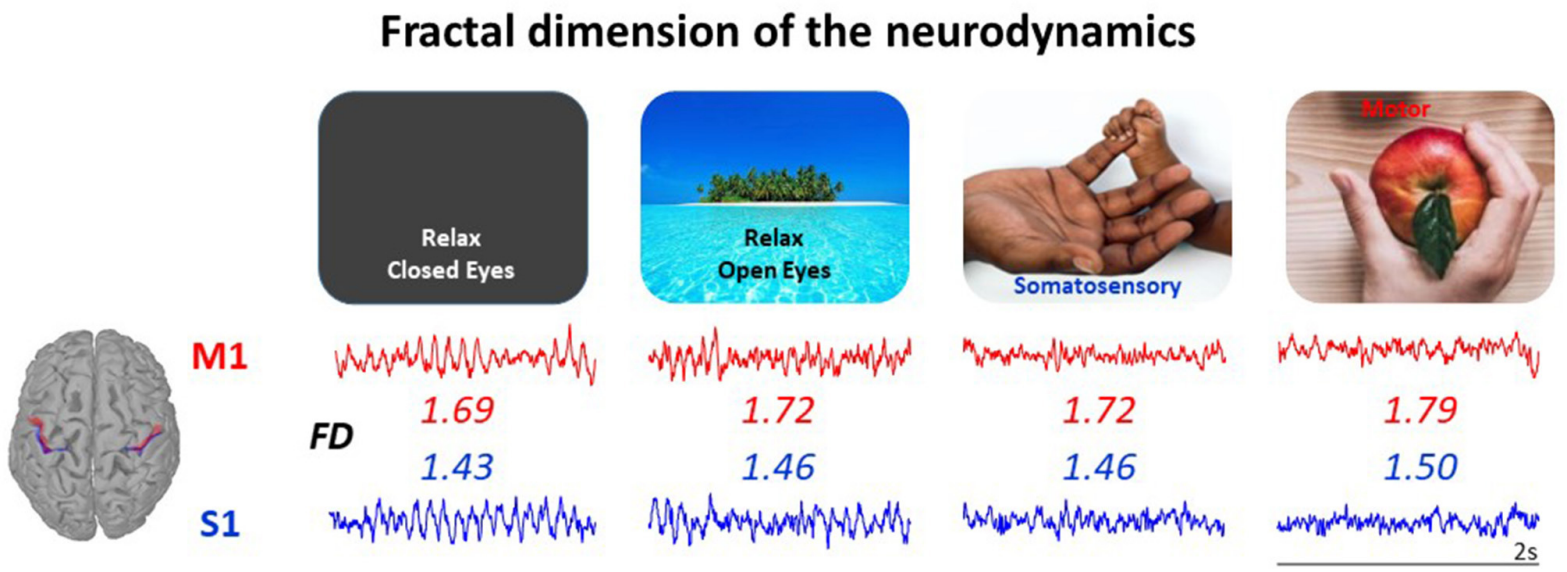
The neurodynamics complexity measured via its fractal dimension (FD) is a single number enabling to characterize the state of a neuronal network node, even at rest. FD of the neurodynamics (2 s in each state) increases when passing from relax in absence of any stimuli **(Left)** to selective sensory perception, to active sensorimotor control **(Right)**. The FD of a node mirrors its structural specificity: here, the primary somatosensory hand area (S1, blue) has smaller FD than primary motor hand area (M1, red) in all network states. Both the state-dependency and the cortical district-dependency are statistically significant in the 20 healthy volunteers' population, as reported in Cottone et al. (2017), where the data come from.

The findings from our laboratory support this hypothesis. We observed that the fractal dimension (FD) of EEG signals successfully senses the modulation of the brain activity in physiological conditions, related to aging (Zappasodi et al., 2015; Smits et al., 2016), circadian rhythm (Croce et al., 2018), behavioral states (Cottone et al., 2017) and neuronal networks' functional role (Marino et al., 2019), and the alterations of the brain activity in clinical conditions (Zappasodi et al., 2014; Smits et al., 2016; Porcaro et al., 2019). Notably, beyond being sensitive to the networks' state, FD offers a tool to parcel the cortex on the base of the local neurodynamics, complementing the Brodmann's cytoarchitectonics criterion (Cottone et al., 2017) (Figure 1).

## Neuronal Network Spoken Language and Electroceuticals

Nowadays the ability to develop therapeutic procedures by intervening on the body physiology by electric signals gives rise to the innovative branch in the medical field: the Electroceuticals (Reardon, 2014). Parallel to the need for technological advancements, they require further knowledge about the correct signals to be provided to the appropriate targets. We propose here a hypothesis on this matter, in the case of neuromodulation, the change of neuronal excitability.

By linking theoretical and experimental studies, the neuroscientific community is revealing network dynamics properties attuned with FeeSyCy mechanisms (Destexhe and Marder, 2004; Deco et al., 2011) that inspired our model of communication within neuronal networks. The model states that every NN—were nodes can be made of neurons, groups of neurons or wider brain regions—develops a “language” shared by its nodes made of exchanged electric pattern, which dynamics' shape brings information (word, Neuronal Network Spoken Language). Notably, when assessing the fractal dimension of the bipolar EEG whole-brain signals we sensed phenomena sensed even by other measures. Noteworthy, when we assessed local neuronal ensemble neurodynamics, the fractal dimension, and not other measures, sensed in resting-state tiny changes with clinical relevance (Porcaro et al., 2019).

The neuroscientific community states that the efficacy of neuromodulation, the change of neuronal electric excitability, depends on the frequency of the stimulation in a region-dependent manner (Brinkman et al., 2016; Fusco et al., 2018), revealing that the intrinsic dynamics of the stimulation target enhances neuromodulation capability. In a seminal non-invasive transcranial electric stimulation (tES) study (Cottone et al., 2018), we proved that a current which mimics the endogenous dynamics of the target neuronal pools, neuromodulates more efficiently than the sinusoid at a locally-tuned frequency, suggesting that structured patterns transmit entrainment more than a non-structured stationary signal.

Near and more long-term future will see further electroceutical personalizations, by developing tools to “speak” the neuronal network language, thus better tuning the neuromodulation to the desired neuronal pool target and obtaining higher efficacy in compensating symptoms secondary to alterations of the neurodynamics, like depression, addiction, pain, fatigue.

This nature of the body-brain in continuous adaptive communication with the environment makes a continuously changing structure that is “to be is to become”.

## Author Contributions

FT conceived the paper and supervised the writing. FT and FZ contributed to the writing of the original draft. MB contributed to figures creation. MB, TL, EG, and LP contributed to the writing and the editing of the manuscript. All authors reviewed and approved the final manuscript.

## Conflict of Interest

The authors declare that the research was conducted in the absence of any commercial or financial relationships that could be construed as a potential conflict of interest.
